# Directed Pancreatic Acinar Differentiation of Mouse Embryonic Stem Cells via Embryonic Signalling Molecules and Exocrine Transcription Factors

**DOI:** 10.1371/journal.pone.0054243

**Published:** 2013-01-17

**Authors:** Fabien Delaspre, Mohammad Massumi, Marta Salido, Bernat Soria, Philippe Ravassard, Pierre Savatier, Anouchka Skoudy

**Affiliations:** 1 Cancer Research Program, Institut Hospital del Mar d’Investigacions Mèdiques (IMIM), Biomedical Research Park, Barcelona, Spain; 2 CABIMER, Sevilla, Spain; 3 CIBERDEM, Instituto de Salud Carlos III, Madrid, Spain; 4 ICM, Biotechnology and Biotherapy, Paris, France; 5 Stem Cells and Brain Research Institute, Bron, France; 6 Université de Lyon, Lyon, France; Baylor College of Medicine, United States of America

## Abstract

Pluripotent embryonic stem cells (ESC) are a promising cellular system for generating an unlimited source of tissue for the treatment of chronic diseases and valuable *in vitro* differentiation models for drug testing. Our aim was to direct differentiation of mouse ESC into pancreatic acinar cells, which play key roles in pancreatitis and pancreatic cancer. To that end, ESC were first differentiated as embryoid bodies and sequentially incubated with activin A, inhibitors of Sonic hedgehog (Shh) and bone morphogenetic protein (BMP) pathways, fibroblast growth factors (FGF) and retinoic acid (RA) in order to achieve a stepwise increase in the expression of mRNA transcripts encoding for endodermal and pancreatic progenitor markers. Subsequent plating in Matrigel® and concomitant modulation of FGF, glucocorticoid, and folllistatin signalling pathways involved in exocrine differentiation resulted in a significant increase of mRNAs encoding secretory enzymes and in the number of cells co-expressing their protein products. Also, pancreatic endocrine marker expression was down-regulated and accompanied by a significant reduction in the number of hormone-expressing cells with a limited presence of hepatic marker expressing-cells. These findings suggest a selective activation of the acinar differentiation program. The newly differentiated cells were able to release α-amylase and this feature was greatly improved by lentiviral-mediated expression of Rbpjl and Ptf1a, two transcription factors involved in the maximal production of digestive enzymes. This study provides a novel method to produce functional pancreatic exocrine cells from ESC.

## Introduction

Pluripotent embryionic stem cells (ESC) derived from the inner mass of the pre-implanted embryos have the ability to self-renew indefinitely *in vitro* and in appropriate conditions can be enforced to differentiate into a diversity of specialized cell types. Recently, it has been shown that endodermal cell derivatives from ESC can be generated through the *in vitro* recapitulation of major developmental signalling pathways occurring *in vivo* [1]. For instance, a conserved mechanism for mesoderm-endoderm lineage commitment involves Nodal, a TGFβ family member, and can be mimicked *in vitro* by activin A, yielding a high percentage of endodermal-like cells [2], [3], [4]. From this cell population, different studies have used instructive signals playing a role in pancreatic organogenesis and β-cell differentiation to commit ESC to similar fates *in vitro* in order to obtain a source of replaceable β-cells for diabetic patients [5], [6], [7]. In addition to the endocrine compartment, the pancreas is composed by exocrine cells including ductal and acinar cells. Acinar cells are responsible for the synthesis of secretory digestive enzymes, and alterations in the acinar differentiation program have been linked to exocrine pancreatic diseases, such as chronic pancreatitis and adenocarcinoma [8]. Therefore, providing normal *in vitro* models of acinar differentiation from ESC could be helpful to understand better these processes as primary acinar cultures fail to retain a differentiated phenotype [9], [10]. We previously demonstrated the generation of acinar cells from mESC on the basis of the genetic selection of elastase 1 (Ela1)-producing cells and the differentiation with conditioned medium from the culture of fetal pancreatic tissues [11]. As this medium contains signals that also promote the differentiation of other pancreatic cell lineages, the isolation of the acinar-like cells was required. In this sense, one important aspect missing in many pancreatic differentiation protocols is to assess the extent of selectivity in cell lineage induction. In this regard, other studies have reported the expression of acinar markers from ESC by manipulating several developmental pathways already established for endocrine differentiation or without examining their role on endocrine gene expression [12], [13], [14], [15]. Therefore, progress in the knowledge of how acinar cells are formed during embryogenesis is essential for the improvement of strategies assessing ESC exocrine differentiation.

Pancreatic organogenesis is a highly regulated process controlled by the gut microenvironment that orchestrates the expression of key transcription factors that, in turn, specify the different pancreatic cell types [16]. Both endocrine and exocrine cells derive from a common pool of progenitors present in the foregut endoderm. The cross-talk between several pathways including the inhibition of Shh and RA signalling activation specifies the pancreatic domain at early stages and regulates the emergence of Pdx1-expressing progenitors that can be expanded by FGF10 [17], [18], [19], [20]. In addition, Ptf1a is a bHLH protein essential for pancreatic formation and in its absence pancreatic progenitors assume an intestinal fate [21], [22]. Gradual reduction of Ptf1a dosage in mice leads to pancreatic hypoplasia and delayed exocrine cytodifferentiation [23]. In the adult, Ptf1a is only expressed in acinar cells as a component of PTF1, a heterotrimeric transcriptional complex including a ubiquitous E-protein and Recombination signal-binding protein J–like (Rbpjl) [24], [25], [26]. During early pancreatic development, Ptf1a requires the interaction with the Rbpj isoform for pancreatic growth and morphogenesis. Then at the onset of acinar cell development, Rbpj is replaced by pancreas-restricted Rbpjl, which confers a higher transcriptional activity to PTF1 leading to maximal expression of secretory digestive enzymes [27], [28].

The aim of this study was to explore alternative routes of exocrine ESC differentiation. To this purpose: *a*) we sequentially activated the *in vivo* pancreatic patterning signals using previously described molecules for the formation of endodermal and pancreatic progenitors together with a new combination of molecules aimed at the generation of exocrine progenitors and, *b*) we enforced Ptf1a and Rbpjl expression using lentiviral gene transduction. Using this strategy, we demonstrated that the modulation of FGF, follistatin and glucocorticoid signalling pathways, which are known to influence exocrine differentiation *in vivo* [16], [29], promoted the generation of acinar progenitors from endodermal-like cells in an efficient and selective manner. When this protocol was coupled to high expression of Ptf1a and Rbpjl (designed Ptf1a^High^ and Rbpjl^High^), an important rise in the expression of digestive enzymes was observed and cells became responsive to secretagogues. We believe that this new protocol is improved to others in that: i) it favours the generation of exocrine progenitors over the production of the endocrine lineage, ii) it leads to a more mature pattern of the regulatory digestive enzyme expression modules than other reported protocols [11], and iii) generates functional cells in a shorter time [11], [13], [14], [15]. Therefore, this approach might be instrumental for a gain of knowledge of the developmental acinar program and the establishment of *in vitro* models for the research on pancreatic exocrine disease.

## Materials and Methods

### ESC Culture and Differentiation Conditions

Undifferentiated CGR8 ESC were cultured in Glasgow’s modified Eagle’s medium (GMEM, Gibco) supplemented with 10% foetal bovine serum (FBS), 0.1 mM 2-mercaptoethanol, 1 mM sodium pyruvate,1% non-essential amino acids (Gibco), 2 mM glutamine, 1% penicillin–streptomycin, and 1000 units/ml LIF as previously reported [30].

**Figure 1:**
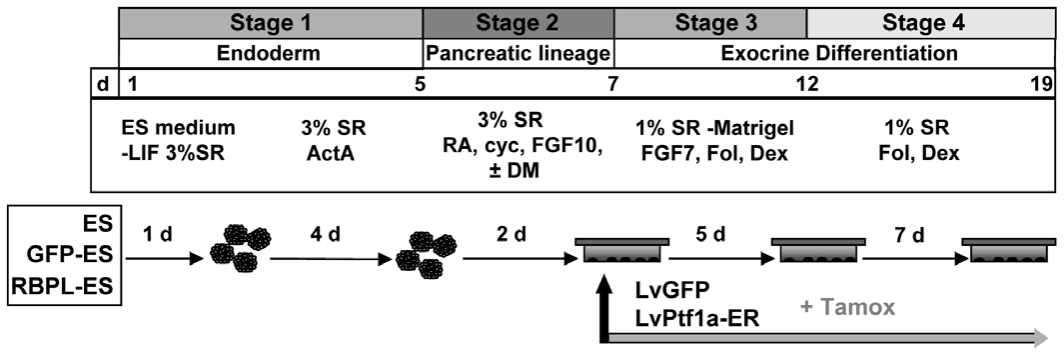
Protocol for pancreatic acinar differentiation. ESC were induced to differentiate during 19 days though 4 sequential stages including the generation of definitive endoderm and pancreatic progenitors as well as the formation and expansion of acinar progenitors. d (days); ActA (activin A); Cyc (cyclopamine); DM (dorsomorphin); Fol (follistatin); Dex (dexamethasone). In some experiments, ESC lines stably expressing GFP or Rbpjl were differentiated throughout the protocol. At the end of stage 2, cells were infected with a lentivirus expressing an ER-fused Ptf1a construct or GFP as control, and treated daily with Tamox until the end.

To initiate differentiation, mouse ESC (mESC) were aggregated in suspension (3.4×10^4^ cells/ml) in ESC medium supplemented with 3% Knockout Serum Replacement (SR) without LIF for 1 day. To generate pancreatic progenitors, 100 ng/ml activin A (R&D Systems) was added to this medium and embryoid bodies (EB) further grown during 5 days, with fresh activin A replacement every 2 days. Subsequently, cells were cultured in medium supplemented with 3% SR, 50 ng/ml FGF10 (Sigma), 60 ng/ml RA (Sigma), 8.2 ng/ml cyclopamine (Toronto Research chemicals) and 0.8 ng/ml dorsomorphin (DM) (Biomol Internat.) for 2 additional days. EB were then plated in gelatin or Matrigel (BD Biosciences) coated tissue dishes in 1% SR supplemented medium. To enforce exocrine differentiation, cells were incubated after 12 hours with 6.2 ng/ml follistatin (Sigma), 50 ng/ml FGF7 (Sigma), 39 ng/ml dexamethasone (Sigma), for 5 days and subsequently treated with follistatin and dexamethasone at the same concentration for an additional week (Fig. 1). For lentiviral gene transduction in differentiating ESC, EB were infected at a multiplicity of infection 1∶10 with lentivirus expressing GFP (LvGFP) or the Ptf1a-ER fusion construct (see above). Medium was changed on the next day and supplemented with 2 µM 4-hydroxytamoxifen (Tamox) (Sigma) (Fig. 1).

### Lentiviral Vector Production and Gene Transduction

*pGAE-CAG-eGFP/WPRE* harbours the sequence encoding the enhanced green fluorescent protein driven of the cytomegalovirus/chicken β-actin fusion (CAG) promoter. *pCAG-Rbpjl* was generated using the Gateway clonase technology using the *pCMV-Rbpsuh-l* plasmid provided by R. Macdonald (University of Texas, Southwestern Medical Center, Dallas, TX) and the 2K7 shuttle vector, containing the PGK promoter and the *bla* ^R^ selectable gene.

Methods for producing lentiviral particles in 293 cells have been described elsewhere [31]. Briefly, 293T cells were transfected using lipofectamine or lipofectamine plus (Invitrogen) with mixtures of DNAs containing the *pGRev* or *pMD2G* plasmids encoding the VSV-G envelope, the *pSIV3+* or *p8.9* plasmids encoding the encapsidation proteins, and the *pCAG-Rbpjl* or *pTRIP* plasmid expressing the Ptf1a fused to the mutated estrogen receptor ligand-binding domain (ERT2) under the control of the cytomegalovirus promoter [32]. On the next day, cells were refed with DMEM and cultured for a further 24 hours. The supernatant was collected, centrifuged, and filtered to remove cell debris.

For infection of undifferentiated ESC with lentivirus expressing GFP or Rbpjl, cells were plated at a density of 10^4^ cells in 24-well plates in 1 ml of medium composed of 100 µl of ESC medium and 900 µl of culture supernatant from virus-producer cells with polybrene (Sigma) (6 µg/ml). After 48 hours, ESC were trypsinized, plated out at 10^4^ cells per 10-mm tissue culture dish and maintained in complete or blasticidin supplemented ESC medium for 6 days. Cell colonies were then individually amplified and characterized for transgene expression.

### Quantitative RT- PCR Analysis

Total RNA was prepared using the GenElute mammalian total RNA kit (Sigma) and treated with DNase I (Ambion). Real time RT-PCR was performed in triplicate on an ABI Prism 7900HT Sequence Detector using the TaqMan RT reagents (Applied Biosystems) for retrotranscription, the quantitative SYBR Green PCR kit (Applied Biosystems), and the primers shown in Table S1. The data were processed using SDS 2.1 software and results were normalized to Hprt mRNA levels.

### Immunocytochemistry

Cells were fixed with 4% paraformaldehyde in PBS for 15 min and permeabilized with 0.1% Triton X-100 and 0.1% saponin for 20 min. After PBS washes, cells were incubated in 0.1% Tween-20-PBS supplemented with 1% gelatin for 1 hour and further incubated with primary rabbit antibodies against Ptf1a [31], Rbpjl (kind gift from R. Wagener, University of Cologne, Germany), Pdx1 [30], HNF1β (Santa Cruz), α-amylase (Amyl) (Sigma), carboxypeptidase A1 (Cpa1) (Biogenesis), glucagon (Gluc) (Dako), rat liver glycogen synthase (Gys2) (Sigma-GenoSys), mouse antibodies against chymotrypsinogen (Chymo) (Biogenesis), guinea-pig antibodies against insulin (Ins) (Dako) or goat antibodies against α-fetoprotein (Afp) (Santa Cruz). Primary antibody was detected using anti-IgG coupled to Alexa 488, Alexa 555 or Alexa 546 (Invitrogen, Jackson). Nuclear labeling was performed with ToPro-3 iodide or DAPI (Molecular Probes). Immunofluorescence staining and GFP expression were visualized with a Leica TCS-SP2 confocal microscope. For quantification of positive cells, clusters were randomly selected from triplicates of two to three independent experiments and the average value ± SEM was determined.

### Amylase Secretion Assay

Cells were washed with PBS and incubated with fresh cell culture medium without FBS and supplemented or non-supplemented (controls) with 1 pM cholecystokinin octapeptide (CCK) (Sigma) or with 5 µM carbachol for 30 min at 37°C. Culture supernatants were then collected and cells lysed in Krebs-Ringer buffer containing 0.2% BSA. Amylase activity was determined using the Infinity™ Amylase Liquid Stable Reagent (Termo Electron). To normalize the amount of amylase secretion, the total protein content was measured by the Bradford method. Amylase released into the supernatant and amylase content of the cell pellets were determined in triplicates.

### Statistics

Statistical differences were analyzed by the Student’s *t* test. p values as *p<0.1; **p<0.05 and ***p<0.005 were considered statistically significant.

## Results

### Directed Pancreatic Acinar Differentiation of mESC in a Stepwise Fashion through the Regulation of FGF, Follistatin, and Glucocorticoids Signalling Pathways

#### Up-regulated expression of genes marking endodermal and pancreatic cell populations during stages 1–2

**Figure 2:**
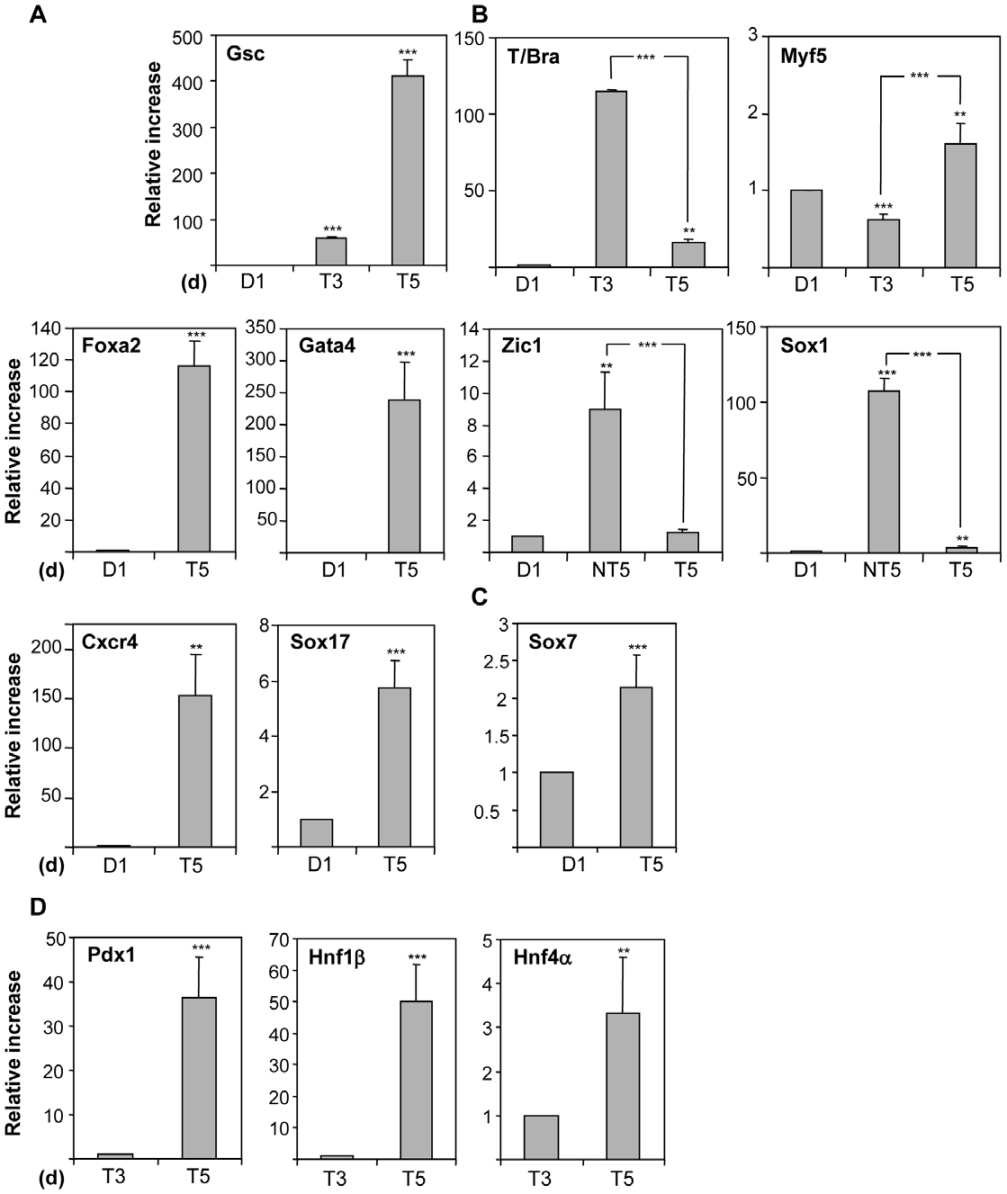
Gene expression of early germ-layer specific markers at stage 1 by qRT-PCR. Cells were induced to differentiate as EB in the presence of 100 ng/ml activin A as indicated in Fig. 1. After 3 or 5 days (stage 1), cultures were harvested and subjected to qRT-PCR analysis for the indicated early germ-layer (A–C) and foregut/pancreatic (D) markers. Histograms show the relative expression levels normalized to the loading control Hprt. Error bars indicate the standard deviations of 4 experiments. (d), days; T, treated cells; NT, non-treated cells. In A–C, p is calculated as compared to D1 and in D, as compared to T3. In D, the expression was analyzed from day 3 onwards as some of the markers were undetectable at day 1.

Our aim was to analyze the ability of endoderm enriched ESC populations to respond to specific signals involved in pancreatic development *in vivo*, using culture conditions previously established to drive mESC into definitive endoderm with minor modifications [33], [34]. ESC were aggregated in suspension for one day in low SR concentration (3%) as EB, to mimic cell interactions occurring *in vivo*. On the next day, EB were treated with 100 ng/ml activin A for up to 4 days to potentiate endoderm specification (stage 1, Fig. 1). We assessed the expression of early germ-layer specific markers by qRT-PCR. This treatment enhanced the expression of *Gsc* and *T/Bra* after 3 days of culture (Fig. 2A–B). At day 5, *T/Bra* was down-regulated while *Gsc* was further enhanced, suggesting that the cell cultures progress through a transient mesendoderm step. Indeed, from day 1 to day 5, the extra-embryonic endoderm marker *Sox7* and the mesoderm marker *Myf5* did not show a marked increase, whereas the neuroectoderm markers *Sox1* and *Zic1* were drastically down-regulated in comparison with non-treated cultures (Fig. 2B–C). By contrast, the definitive endodermal markers *Foxa2*, *Cxcr4*, *Gata4* and *Sox17* were significantly up-regulated in the treated cultures at day 5 (Fig. 2A), indicating an activation of the definitive endoderm differentiation program. In agreement with endoderm and primitive gut formation, evidenced by the increase in *HNF1*β and *HNF4α* (Fig. 2D), the levels of mRNAs encoding for pancreatic *Pdx1* were already strikingly enhanced (Fig. 2D), suggesting the activation of a posterior foregut differentiation program.

**Figure 3:**
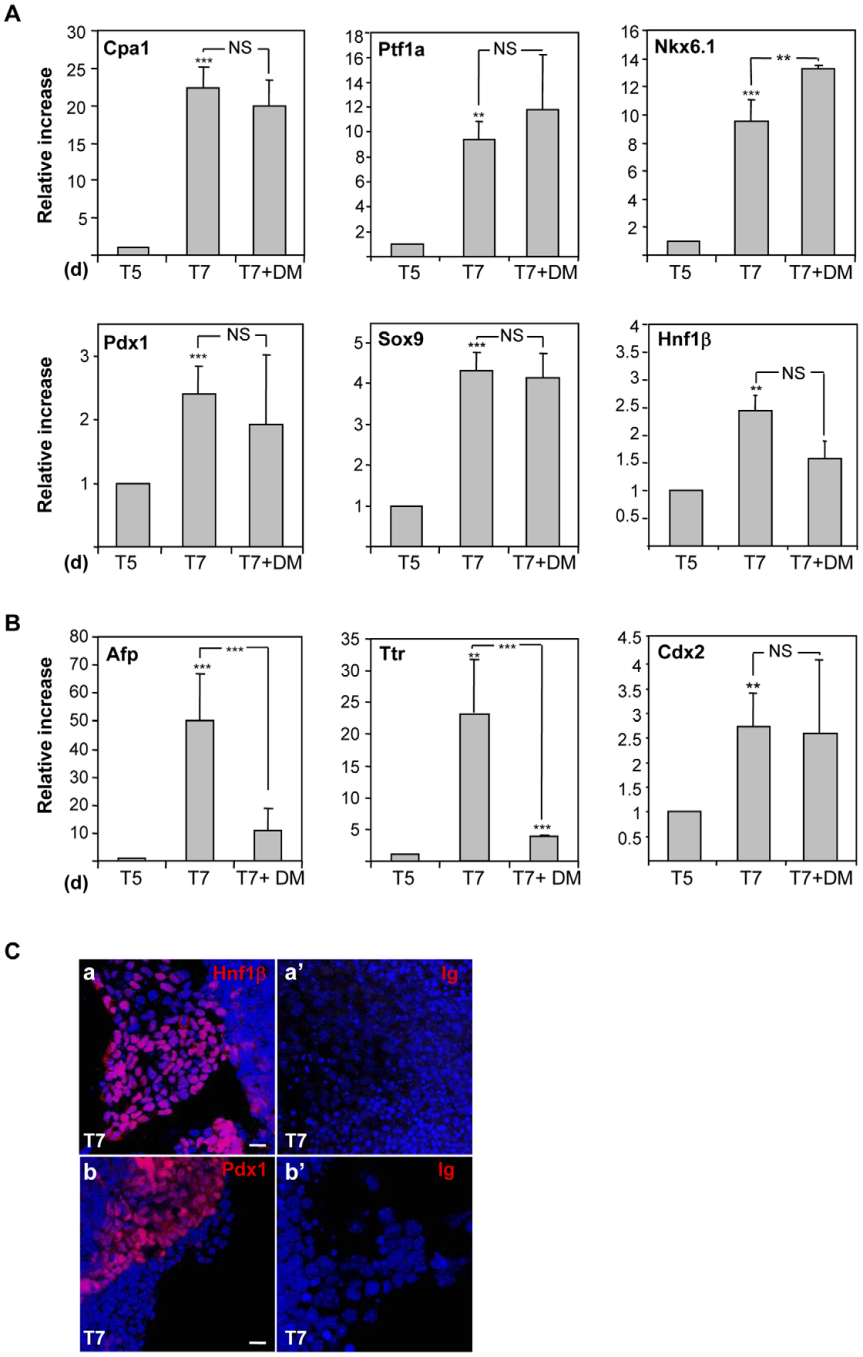
Expression of pancreatic progenitor markers at stage 2. A) Cells were induced to differentiate through progression of stages 1 and 2 with or without DM as indicated in Fig. 1. After 5 (stage 1) or 7 (stage 2) days, cultures were harvested and subjected to qRT-PCR analysis for the indicated markers. Histograms show the relative expression levels normalized to the loading control Hprt. Error bars indicate the standard deviations of 3 experiments. p values correspond to comparisons between T5 and T7 and between T7 and T7+ DM. NS, not significant. The effect of the pro-pancreatic agents was analyzed at the beginning and the end of the corresponding stage. B) Expression of non-pancreatic markers by qRT-PCR as indicated in A. C) Immunofluorescent staining for HNF1β (a) and Pdx1 (b) in 7-day cultures (stages 1 and 2), 12 hours after plating. Nuclei were stained in blue. The corresponding negative controls are shown in (a’) and (b’). Scale bars, 10 µm.

To further promote pancreatic specification, activin A-treated EB were next incubated in suspension during 2 days with FGF10, RA, the hedgehog-signalling inhibitor cyclopamine as previously described [5] and DM, a small-molecule inhibitor of BMP signalling (stage 2, Fig. 1) [35]. As expected, qRT-PCR analysis showed a continued increase in *Pdx1* and *HNF1*β expression during this stage in parallel with a rise of a panel of pancreatic progenitor markers, such as *Cpa1*, *Sox9*, *Nkx6.1,* and *Ptf1a*, which indicates the production of a pancreatic lineage specific cell population (Fig. 3A). The addition of DM at this stage strongly down-regulated hepatic markers (*Afp* and *Ttr*) (Fig. 3B), which is consistent with the requirement of BMPs for the development of the hepatic cell lineage [36], [37]. By contrast, pancreatic markers or *Cdx2* (a marker of the midgut/posterior gut) remained mostly unchanged by the treatment (Fig. 3A). Immunofluorescent stainings confirmed the presence of cells expressing Pdx1 and Hnf1β in these cultures (about 20% and 38%, respectively) (Fig. 3C), further suggesting the generation of pancreatic progenitor cells.

#### Directed generation of pancreatic acinar progenitors (steps 3 and 4)

Next, we plated stage 2-EB in gelatin or Matrigel-coated tissue dishes and incubated them with a combination of factors activating specifically developmental exocrine signalling pathways *in vivo*. Some of those signals regulate the proportion of exocrine versus endocrine cells. For instance, follistatin promotes the growth of exocrine tissue at the expense of endocrine cells and dexamethasone, a glucocorticoid agonist, favours acinar differentiation *in vitro* and *in vivo* [38], [39], [40]. Pancreatic culture explants assays demonstrated a role of FGF7 on exocrine cell proliferation and differentiation [41]. These factors were, therefore, sequentially supplemented during stages 3–4 as described in the Methods section and shown in Fig. 1.

**Figure 4:**
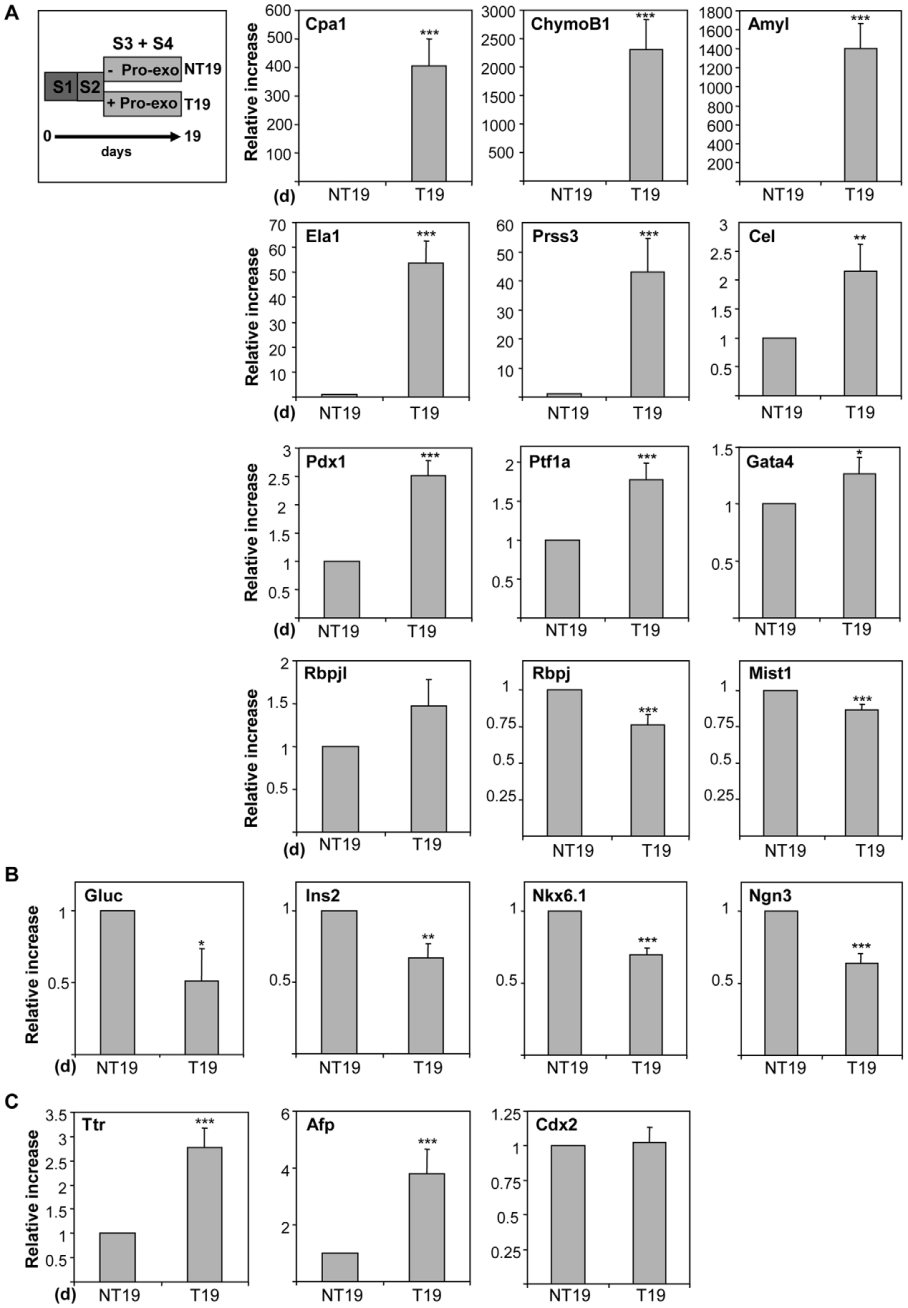
Expression of pancreatic differentiation markers by qRT-PCR at stage 4. Cells were induced to differentiate through-out the whole protocol as indicated in Fig. 1 and analyzed by qRT-PCR for the expression of exocrine (A), endocrine (B) or hepatic (C) markers. Histograms show the relative expression levels normalized to the loading control Hprt. Error bars indicate the SEM of at least 3 experiments. Marker expression at day 19 (T19) was compared to non-treated cells after stage 2 induction and cultured during the same period of time (NT19) to specifically study the effect of the pro-exocrine molecules. p, as compared to NT19.

The cells cultured through stages 1–2, and subsequently treated with pro-exocrine soluble factors until day 19 (T19, whole protocol) or not treated (NT19) (Fig. 4A), were analyzed for the expression of an extended panel of pancreatic markers by qRT-PCR. A prominent induction of mRNA transcripts encoding for digestive enzymes was observed (*Cpa1*, *Amyl* and *ChymoB1*) in T19 cultures as compared to NT19 (Fig. 4A). It should be noted that this induction was even more dramatic if T19 cultures are compared with cells maintained only in 1% SR during the same period of time (SR19) (Fig. S1A). This indicates that transiting through stages 1–2 confers to the cells a higher competence to express spontaneously exocrine markers. In accordance, we observed increased extracellular release of amylase in T19 in comparison with SR19 cultures (Fig. S1B).

The up-regulation of digestive enzyme expression correlated with a discrete to moderate rise of mRNA transcripts encoding for *Ptf1a* and *Gata4*, expressed in acinar progenitors, and *Pdx1*, which cooperates with PTF1 to enhance acinar gene expression and necessary for exocrine development (Fig. 4A) [42], [43], [44]. *Rbpjl* expression was also increased, but the difference was not statistically significant. *Rbpj* mRNA levels were reduced as were those for *Mist1*. These last two genes are expressed in acinar cells but are not pancreas-specific markers [45].

On the other hand, the expression of endocrine markers, including islet hormones insulin 2 (*Ins2*) and glucagon (*Gluc*) and transcription factors marking the endocrine progenitors (*Nkx6.1* and *Ngn3*), was decreased (Fig. 4B). In addition, hepatic *Afp* and *Ttr* were slightly up-regulated in comparison to strong up-regulation for digestive enzymes (Fig. 4C and Fig. S1A) whereas the gut marker *Cdx2* was not modulated (Fig. 4C).

**Figure 5:**
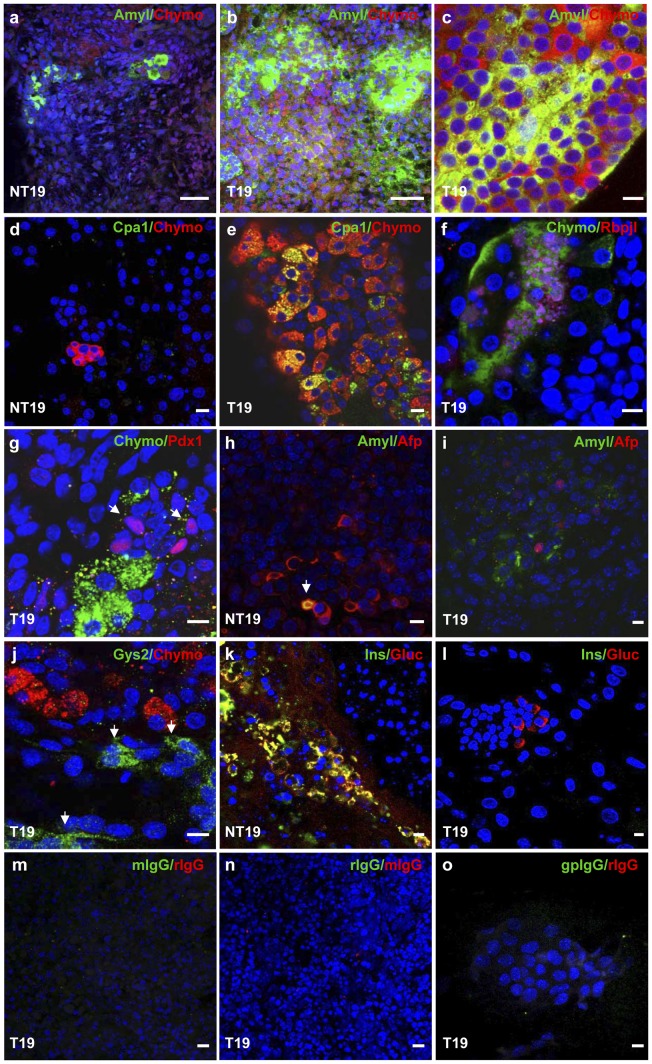
Immunofluorescent analysis of differentiated cell cultures. Staining was performed for Chymo (a–g, j), Amyl (a–c, h–i), Cpa1 (d–e), Rbpjl (f), Pdx1 (g), Afp (h–i), Gys2 (j), Ins (k–l) and Gluc (k–l) in NT19 (a, d, h, k) and T19 cultures (b–c, e–g, i–j, l) as indicated. Nuclei were stained in blue. Negative controls (m–o) were performed with irrelevant antibodies against rabbit (r), mouse (m), goat (g) or guinea pig (gp) as indicated. Scale bars: a–b, 50 µm; c–o, 10 µm.

Expression of selected markers was confirmed by immunofluorescence (Fig. 5). In T19 cultures, large Amyl^+^ and Chymo^+^ cell clusters were found (Fig. 5b–c) as compared to control NT19 cultures (Fig. 5a) (26.5±6.03% in T19 vs 4.9±1.05% in NT19, p<0.05). Also, a large proportion of Chymo^+^ cells co-expressed Cpa1 (Fig. 5e) in comparison with controls (Fig. 5d). In line with qRT-PCR studies, only a subset of these Chymo^+^ cells were also Rbpjl^+^ and were often organized in luminal-like structures (Fig. 5f). Although *Pdx1* mRNA levels were increased in T19 cultures (Fig. 4A), nuclear Pdx1^High^ was observed in cell subgroups expressing low Chymo or being negative for this marker (Fig. 5g), while it was mostly undetectable in cells expressing high levels of the enzyme. This is in agreement with *in vivo* patterns in which only a subpopulation of differentiated acinar cells expresses Pdx1^Low^.

By contrast, very few Gluc^+^ and no Ins^+^ cells were found in the T19 condition (Fig. 5l) whereas they were present in large cell clusters in NT19 cultures (Fig. 5k). Counting assays confirmed a significant reduction in the number of hormone-expressing cells using the whole protocol (15.2±2.5% in NT19 vs 5.2±1.6% in T19, p<0.05). The presence of very few double positive Amyl^+^/Afp^+^ cells was observed in NT19 (Fig. 5h) but not in T19 cultures. Indeed, the few Afp^+^ were essentially excluded from the large Amyl^+^ cell clusters and were, occasionally, located close to isolated or small groups of Amyl^+^ cells (Fig. 5i). Likewise, no co-expression of Chymo and Gys2, responsible for glycogen synthesis in liver, were found in T19 (Fig. 5j), further suggesting that the generated exocrine cells arise from true pancreatic progenitors. Altogether, we show the development of a protocol for pancreatic differentiation, which favours the production of acinar progenitors over the generation of both pancreatic endocrine and hepatic lineages.

### *In vitro* Differentiation of Transgenic RBPL-ES Cells: Cooperation with Ptf1a for Maximal Production of Digestive Enzymes

The fact that the exocrine transcription factor *Rbpjl* and a specific set of secretory enzymes, including *Trypsin 3 (Prss3)*, *Carboxyl ester lipase (Cel)* and *Ela1*, induced at later stages of embryonic development [28] were increased at lower levels with the T19 protocol, suggested an incomplete activation of the exocrine differentiation program (Fig. 4A).

**Figure 6:**
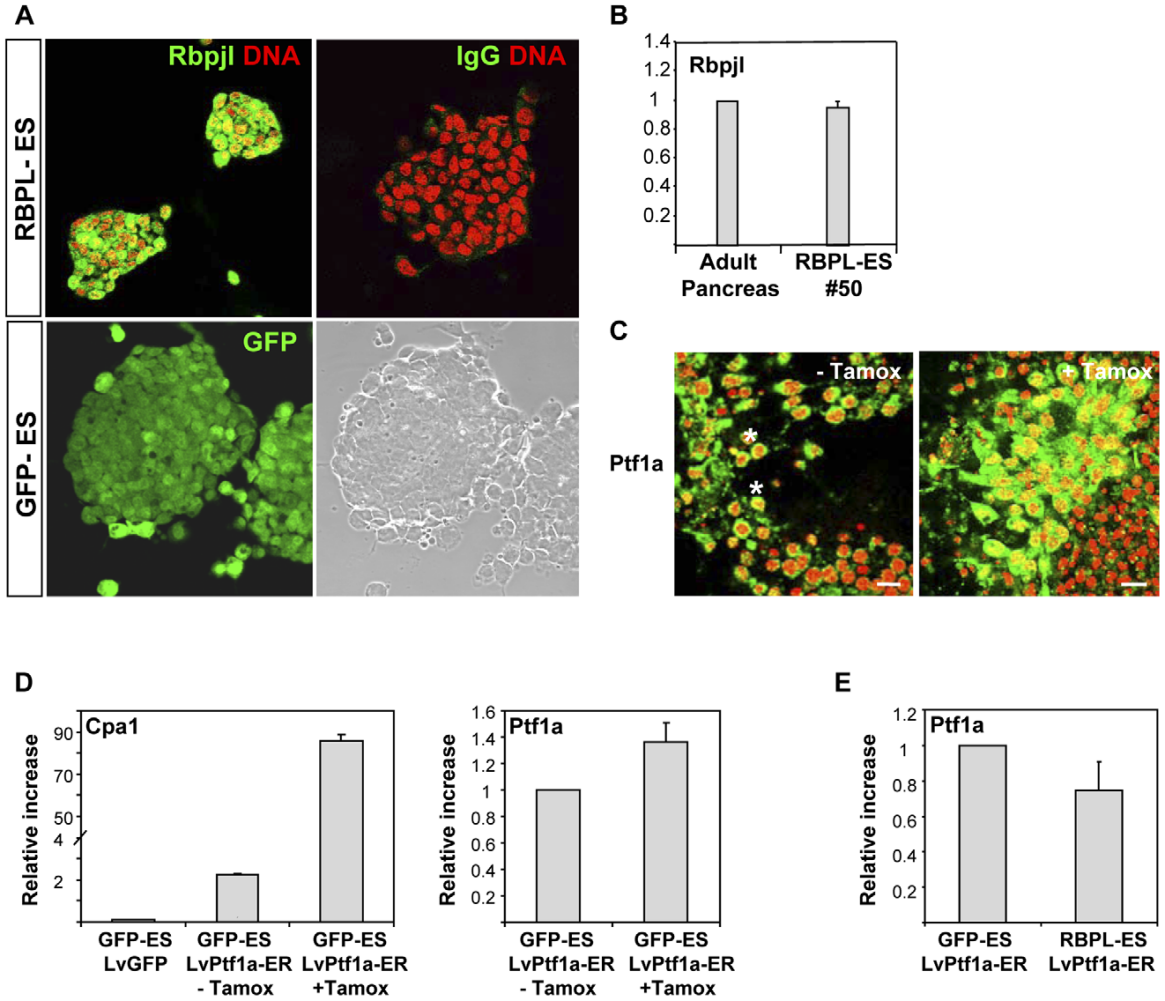
Characterization of transgene expression in undifferentiated and differentiated ESC lines. A) Analysis of transgene expression in RBPL-ES. Undifferentiated RBPL-ES were stained by immunofluorescence with an anti-Rbpjl antibody or an irrelevant one (green) and with Tropo3 (red) to label nuclei. GFP expression in GFP-ES cells was analyzed by confocal microscopy. The engineered ESC lines displayed a normal karyotype and retained their self-renewal capacity (not shown). Scale bars, 50 µm. B) Rbpjl mRNA levels of clone # 50 were comparable to those of mouse adult pancreas by qRT-PCR. C) Immunofluorescence analysis of Ptf1a expression and relocalization in differentiating ESC infected with Lv-Ptf1a-ER and treated with DMSO (−) or with Tamox (+), two days after. Ptf1a expression is shown in green while the nuclei are stained in red. Asterisks (*) show nuclear Ptf1a staining in cells non-exposed to Tamox. Scale bars, 10 µm. D) qRT-PCR analysis of Cpa1 expression in GFP-ES cells differentiated through the protocol until the end of stage 3. Cells were infected with a control LvGFP or the Lv-Ptf1a-ER and incubated with or without Tamox. Ptf1a mRNA expression is also shown as an indicator of LvPtf1a-ER gene transduction. E) qRT-PCR analysis of ectopic Ptf1a mRNA expression at the end of the protocol in GFP-ES and RBPL-ES cultures infected with LvPtf1a-ER.

To assess the ability of Rbpjl in regulating digestive enzyme gene modules to achieve advanced differentiation, lentiviral gene transduction was used to generate ESC cell lines with stable expression of Rbpjl (RBPL-ES). An ESC line expressing GFP was used as control (GFP-ES) (Fig. 6A). Characterization of transgene expression in RBPL-ES by RT-PCR, Western blot (not shown), and immunofluorescence confirmed the correct expression of ectopic Rbpjl with a nuclear localization (Fig. 6A). ESC clones expressing the highest *Rbpjl* mRNA levels as compared to adult pancreas were selected by qRT-PCR, such as clone #50, and behaved similarly in differentiation studies (Fig. 6B, and data not shown). Because the major known role of Rbpjl is as a Ptf1a transcriptional partner, the effect of superimposed Ptf1a overexpression during ESC exocrine differentiation *in vitro* was analyzed in parallel. We used a lentivirus expressing the Ptf1a gene fused to the mutated estrogen receptor ligand-binding domain (LvPtf1a-ER), allowing an inducible nuclear Ptf1a expression after Tamox addition. LvPtf1a-ER or LvGFP, as control, were introduced at the exocrine progenitor stage (stage 3, Fig. 1), to mimic more closely the timing when Ptf1a levels start to increase during pancreatogenesis. Transgene expression was observed in approximately 50% of ESC and addition of 2 µM Tamox was sufficient to relocate Ptf1a into the nuclear compartment (Fig. 6C). After 5 days of differentiation, control GFP-ESC infected with LvPtf1a-ER and exposed to Tamox expressed higher levels of *Cpa1* mRNAs as compared with the same DMSO-treated cells (Fig. 6D). Of note, these later cultures (non-treated cells infected with LvPtf1a-ER) exhibited higher Ptf1a activity as compared to LvGFP infected cells, consistent with detectable nuclear amounts of Ptf1a (Fig. 6C). This likely reflects some leakage in Ptf1a-ER nuclear translocation. Therefore, we studied the conditions in which transgenic ES cells were infected with LvGFP+Tamox versus LvPtf1a-ER+Tamox.

**Figure 7:**
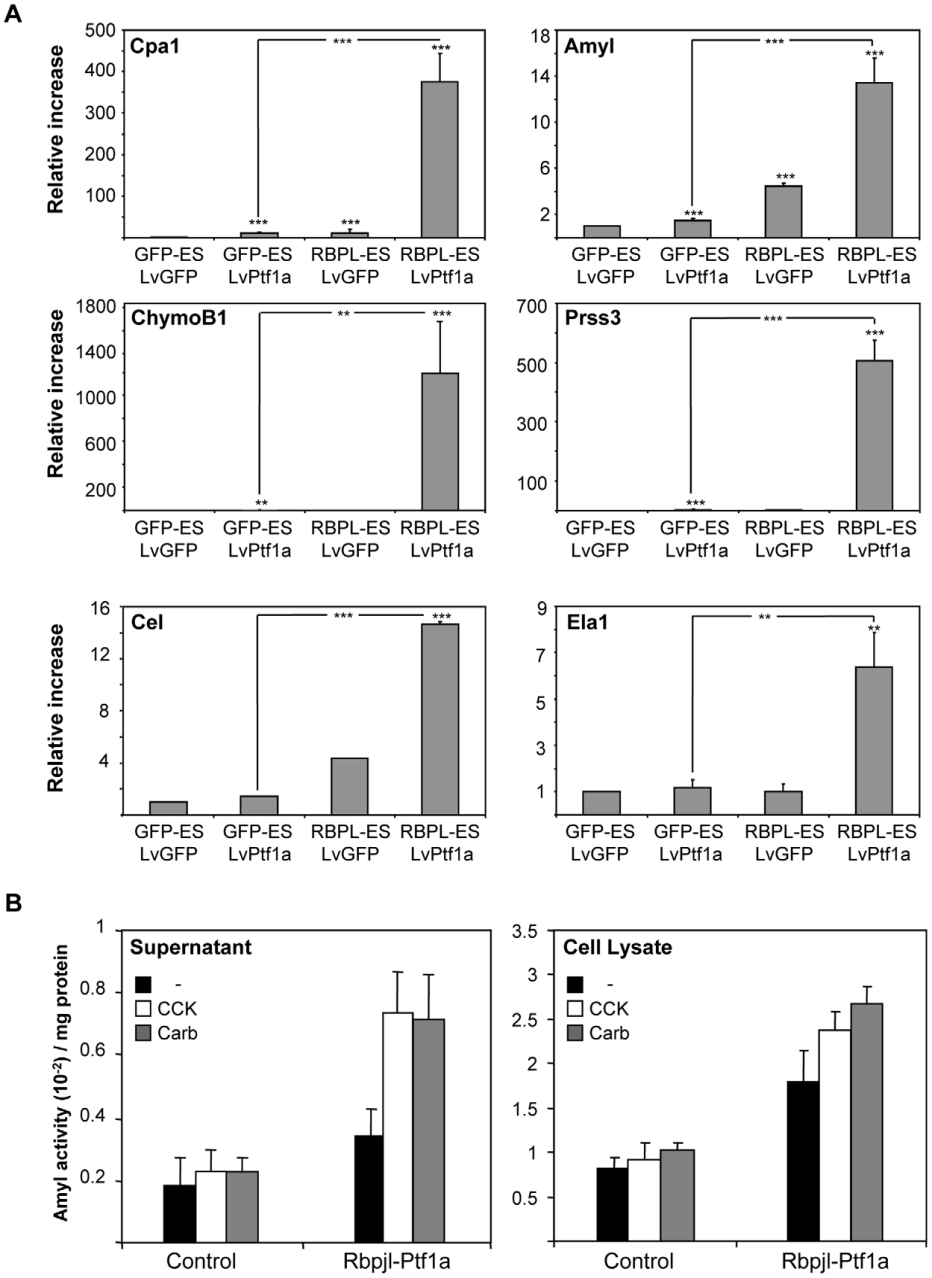
Digestive enzyme gene expression in transgenic GFP-ES and RBPL-ES differentiated throughout the whole protocol. A) Analysis of digestive enzyme gene expression by qRT-PCR at the end of the protocol at the indicated culture conditions. T19 cultures of ESC and GFP-ES infected with LvGFP showed no significant differences in gene expression levels (not shown). Histograms show the relative expression levels normalized to the loading control Hprt. Error bars indicate the standard deviation of 2 experiments performed in triplicates. p, as compared to GFP-ES infected with LvGFP. LvPtf1a indicates in this figure LvPtf1a-ER treated with Tamox. B) Secretagogue-mediated exocytosis in differentiated cells. Control GFP-ES or RBPL-ES cells infected with LvGFP or LvPtf1a-ER, respectively, were differentiated through the whole protocol and stimulated for 30 minutes with CCK and carbachol. Amylase activity was measured in both the supernatant and cell lysates.

Following the whole differentiation protocol and infection with LvGFP (Fig. 1), RBPL-ES cultures showed a limited but significant increase in a subset of secretory enzymes (*Cpa1*, *Amyl*, *ChymoB1*) in comparison with LvGFP-infected GFP-ES cultures (Fig. 7A), while the levels of endodermal and pancreatic progenitor markers at previous stages remained similar in both conditions (not shown). This differential increase in digestive enzymes between both cell lines was much stronger when cells were infected with LvPt1a-ER and treated with Tamox, while exhibiting similar levels of ectopic *Ptf1a* mRNAs (Fig. 6E). Notably, the combined action of Ptf1a and Rbpjl induced not only an increase in early secretory enzyme Ptf1a targets such as *Cpa1*, *Amyl* and *ChymoB1* [11], [28] but also a highly significant increase in *Prss3*, *Cel* and *Ela1* levels, expressed later during embryonic development (Fig. 7A). This indicates a coordinated activation of the acinar differentiation program leading to a more mature expression pattern of acinar digestive enzymes. In addition, enhanced levels of Mist1, involved in terminal differentiation of acinar cells, was observed in cells overexpressing Ptf1a and Rbpjl when compared to control LvGFP-infected GFP-ES cells (Fig. S2) [46]. By contrast, endocrine (except for glucagon) and hepatic markers were not substantially changed in the same conditions (Fig. S2), in line with a role of Rbpjl being restricted to the exocrine compartment [28]. Immunofluorescence assays showed that in LvPtf1a-infected RBPL ES cultures the percentage of cells expressing digestive enzymes was 33.8±6.3 in comparison to 25.7±3.1 in LvGFP- infected GFP cells (Fig. S3). Of note, the number of cells displaying a strong staining for these markers was increased in cultures overexpressing Ptf1a and Rbpjl relative to control ones: for amylase, 38.4±5.3% versus 23.7±4.7% and for Cpa1, 43.4±2.6% versus 22.5±1.3%, p<0.05.

The increase in the acquisition of more differentiated features was also reflected by the ability of differentiated RBPL-ES cells to secrete amylase at physiological concentrations of secretagogues (Fig. 7B). Thus, an increase of amylase activity was present in these cultures, both in the intracellular and extracellular compartments suggesting increased *de novo* synthesis and secretion of digestive enzymes in response to secretagogues, which are two well established functions of these molecules. By contrast, cells that were not subjected to Ptf1a and RBpjl overexpression were unresponsive to the same stimuli (Fig. 7B). Overall, these results indicate the acquisition of functional properties through the activity of the exocrine transcription factors.

## Discussion

In the last years a number of reports have demonstrated the generation of immature pancreatic endocrine cells from ESC, but much less is known about the generation of acinar cells from pluripotent stem cells. Previously, we genetically produced acinar-like cells from mESC but the protocol was time-consuming and the selection strategy could favor the purification of cells displaying higher proliferative ability and less differentiated features [11]. Herein, we aimed to generate exocrine progenitors from mESC following a directed differentiation method. We were interested in enhancing acinar versus endocrine differentiation *in vitro*, using in the first steps pioneer conditions for definitive endoderm and pancreatic progenitor production [2], [14], [33], [34], [47] and sequentially, a new combination of signals involved in exocrine development.

Among the signalling pathways involved in this cell lineage decision, follistatin stimulates the generation of amylase-expressing cells while repressing the formation of insulin-producing cells [38]. Another signal that may enhance acinar differentiation involves glucocorticoids, which promote acinar differentiation in Pdx1-expressing cells at the expense of β-cell proliferation [40]. Moreover, glucocorticoids up-regulate the maturation state of exocrine cells by regulating the expression of amylase, a feature of advanced acinar differentiation and their secretory capability [48]. Therefore, to sustain exocrine differentiation in ESC cultures, cells were simultaneously treated with both factors as it is unlikely that with one single differentiating agent a robust exocrine differentiation would have been achieved [30]. In agreement with the results of Ren et al. [15], dexamethasone was crucial for an optimal induction of digestive enzyme expression but only when added in combination with the other factors. In this respect, co-treatment with follistatin and FGF7 selectively increased the expression of these markers but to a lesser extent (data not shown). However, the previous combination of factors (Activin A+sodium butyrate+dexamethasone) was somewhat quite inefficient resulting in nearly two-fold increase in the induction of digestive enzymes as compared with control cultures [15]. By contrast, in our experiments there was a substantial increase in the efficiency of induction of digestive enzymes (a factor of 10^3^–10^4^ times as an average estimation) with respect to cultures only treated with 1% SR, which by itself is permissive on ESC acinar differentiation [49]. To further enhance this efficiency, we co-supplemented our cultures with T3, a thyroid hormone that selectively promotes acinar cell proliferation and that cooperates with glucocorticoids in regulating secretory enzyme expression [50], [51]. With addition of T3 we did not observe a significant impact on the magnitude of acinar gene expression (data not shown).

Additional detailed studies will be needed to determine the role of individual differentiating factors in our new method presented here; however, a recent study showed that FGF7 is able to regulate acinar differentiation in mESC [14]. Nonetheless, this protocol was also useful for endocrine differentiation, thus missing a more specific cell lineage approach. In this regard, we previously showed that FGF7 induced the expression of both endocrine and exocrine markers in mESC, supporting its role in the expansion rather the differentiation of pancreatic or lineage progenitors [30], [41]. Therefore, the search for more selective combinations of molecules for the generation of exocrine cells remains necessary. In this sense, a valuable contribution of our protocol is that it favours exocrine differentiation over the endocrine phenotype. It is likely that the differentiation agents used herein impinge on the early endocrine commitment of pancreatic progenitors as suggested by a significant decrease of Ngn3 message levels (Fig. 4). Notably, *Nkx6.1* was also down-regulated in line with recent data showing a mutually antagonist action with Ptf1a in directing endocrine versus acinar cell fate choices [52]. In keeping with this, a significant reduction in the number of cells expressing Ins and Gluc was observed (Fig. 5). Remarkably, the majority of endocrine cells co-expressed both hormones in the NT19 condition, which is indicative of an immature differentiation state and compatible with a default differentiation pathway.

An important aspect that has not been previously studied refers to the generally accepted notion that prolonged exposure to glucocorticoids results in the reprogramming of acinar cells into hepatic-like cells [15], [53]. In our study, the drastic increase in digestive enzymes was not accompanied by a strong rise of hepatic markers (Fig. S1A) and the generated acinar progenitors did not express hepatic Afp and Gys2 (Fig. 5), further indicating that the produced cells maintain their pancreatic identity. Moreover, the fact that in our murine model *Cpa1*, *Chymo* and *Amyl* expression was not affected by BMP inhibition (stage 2, Fig. 3A and data not shown) argues against a hepatic origin in our conditions [37].

Although the directed protocol was more selective and improved the level of induction of digestive enzymes compared to our previous methods, the acinar-like cells were still immature. A complementary strategy to soluble factor-induced differentiation for the generation of functional cell types includes the gain of function of regulatory genes playing a key role during *in vivo* embryonic development. Previously, we showed that combined Ptf1a and Mist1 expression favours the acquisition of an acinar phenotype [11]. However, the overexpression of Ptf1a (alone without the other members of PTF1) only resulted in a strong induction of early digestive enzymes (Cpa1, ChymoB1) but not of those reported to be activated at later stages (Amyl, Ela1) [11], [31]. The present findings support that a Ptf1a-Rbpjl complex is required for the acquisition of a mature acinar phenotype. Thus, Ptf1a and Rbpjl alone could moderately regulate the expression of early digestive enzymes but it was when co-expressed that the level of induction increased substantially (Fig. 7A). Importantly, other Rbpjl-dependent secretory enzymes such as *Prss3*, *Cel* and *Ela1* were significantly increased. It should be noted that the level of induction for each of these genes was strikingly similar to what occurs *in vivo.* In this sense, the grade of reduction in *Rbpjl* ^−/−^ mice appears more pronounced for *Prss3>Cel>Ela1* [28]. As occurs *in vivo*, endocrine and hepatic markers were not substantially affected, despite that *Rbpjl* is expressed in islets [26], [28]. Ultimately, a progression in the developmental program was further demonstrated by the ability of the generated cells to become responsive to secretagogues, a hallmark of acinar functionality. This is a property that is not observed in cells differentiated only with soluble factors (Fig. 7B) and that has not been yet demonstrated by other studies [13], [14], [15]. In summary, we report a new method, which substantially recapitulates pancreas development regarding the modulation of the balance between endocrine and exocrine cell differentiation, and can provide important hints into the key transcriptional pathways that delineate exocrine lineage development in ESC.

## Supporting Information

Figure S1 **Efficiency of digestive enzyme expression in cells differentiated through-out the whole protocol.** A) qRT-PCR analysis of exocrine gene expression in T19 cultures was made in comparison with cells incubated in same conditions in the absence of any inducing factor. Cells were therefore only cultured in 1% SR for 19 days. Error bars indicate the standard deviation of 4 experiments. B) Amylase activity in the supernatants of the indicated cell culture conditions. In T19 cultures, cells did not respond to acinar secretagogues (not shown).
(TIF)

Figure S2 **qPCR analysis for exocrine, endocrine and hepatic markers in transgenic GFP-ES and RBPL-ES differentiated through-out the whole protocol.** Histograms show the relative expression levels normalized to the loading control Hprt. Error bars indicate the standard deviation of 2 experiments performed in triplicates. p, as compared to GFP-ES infected with LvGFP. LvPtf1a indicates in this figure LvPtf1a-ER treated with Tamox. NS, not significant.
(TIF)

Figure S3 **Immunofluorescent analysis of digestive enzymes in cultures overexpressing Ptf1a and Rbpjl differentiated through-out the whole protocol.** Staining was performed for Amyl (a) and Cpa1 (b) in red. Nuclei were stained in blue. Negative control (c) was performed with an irrelevant antibody. Scale bars: a–c, 10 µm.
(TIF)

Table S1 **List of primers used for qPCR.**
(TIF)
