# Supplementary figures and images for: Directed Pancreatic Acinar Differentiation of Mouse Embryonic Stem Cells via Embryonic Signalling Molecules and Exocrine Transcription Factors
Source: PLoS One. 2013 Jan 17;8(1):e54243. doi: 10.1371/journal.pone.0054243 (PMC3547908; doi:10.1371/journal.pone.0054243)

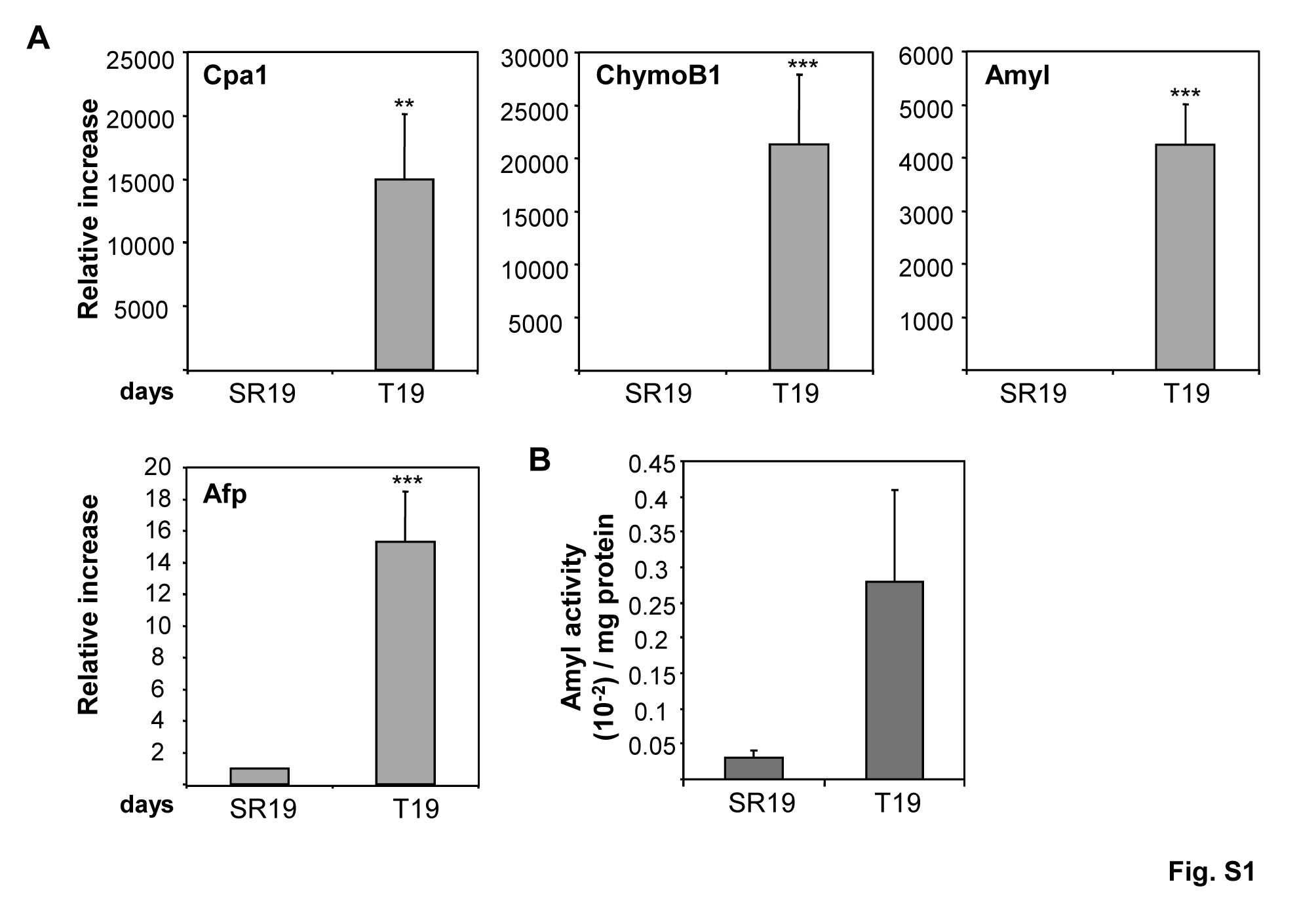

Supplement: Figure S1 — Efficiency of digestive enzyme expression in cells differentiated through-out the whole protocol. A) qRT-PCR analysis of exocrine gene expression in T19 cultures was made in comparison with cells incubated in same conditions in the absence of any inducing factor. Cells were therefore only cultured in 1% SR for 19 days. Error bars indicate the standard deviation of 4 experiments. B) Amylase activity in the supernatants of the indicated cell culture conditions. In T19 cultures, cells did not respond to acinar secretagogues (not shown). (TIF) [file pone.0054243.s001.tif]

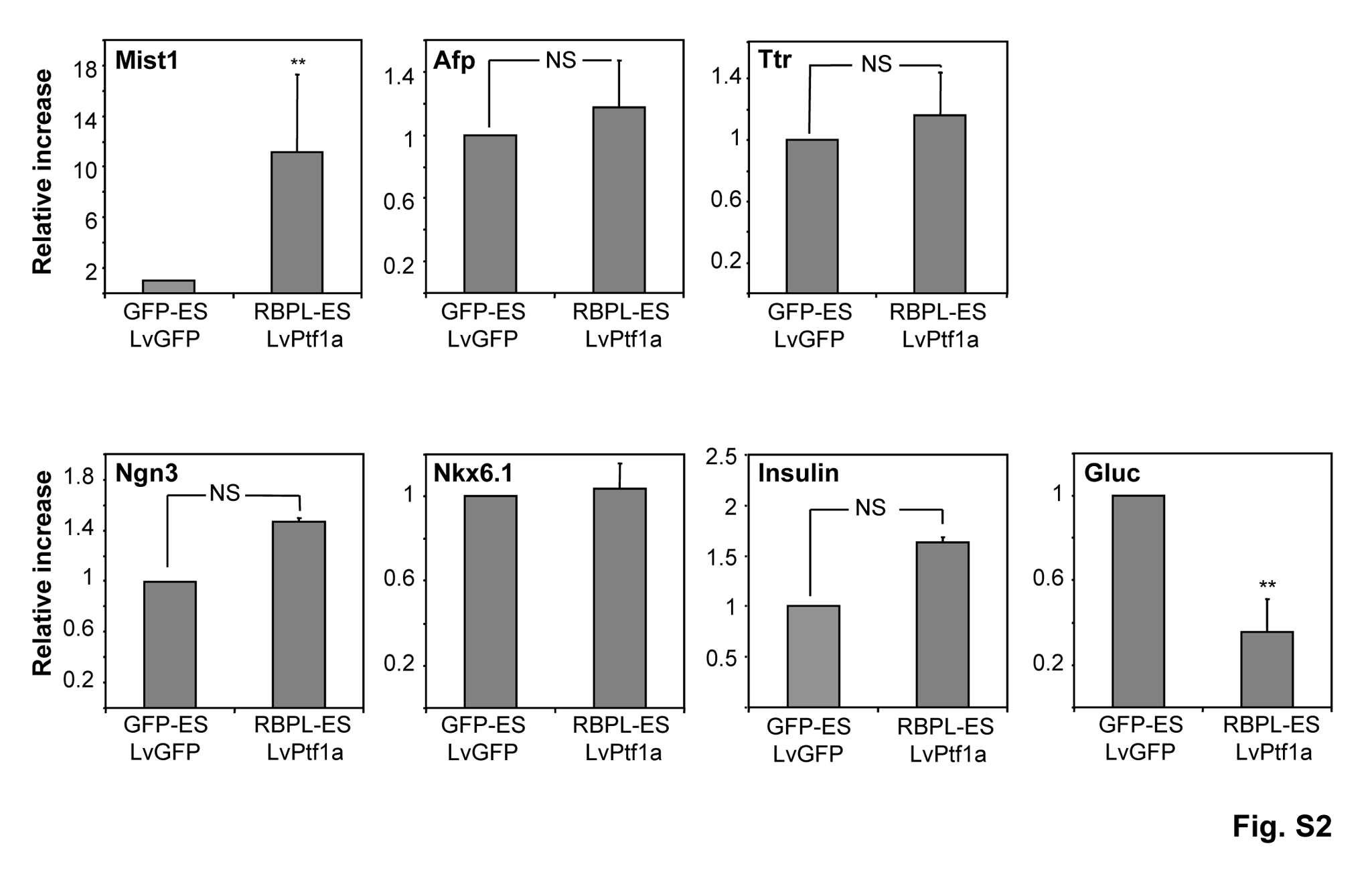

Supplement: Figure S2 — qPCR analysis for exocrine, endocrine and hepatic markers in transgenic GFP-ES and RBPL-ES differentiated through-out the whole protocol. Histograms show the relative expression levels normalized to the loading control Hprt. Error bars indicate the standard deviation of 2 experiments performed in triplicates. p, as compared to GFP-ES infected with LvGFP. LvPtf1a indicates in this figure LvPtf1a-ER treated with Tamox. NS, not significant. (TIF) [file pone.0054243.s002.tif]

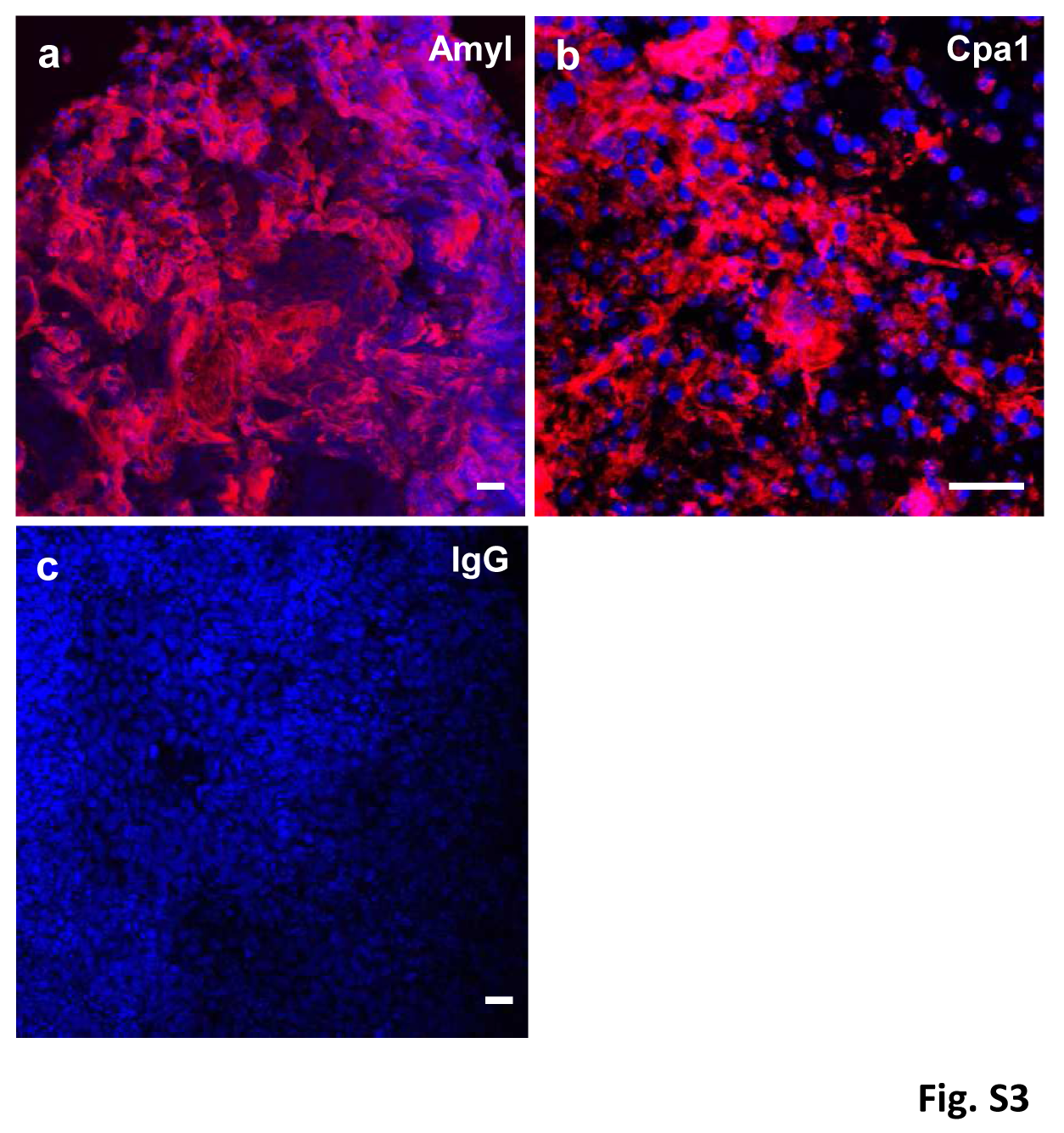

Supplement: Figure S3 — Immunofluorescent analysis of digestive enzymes in cultures overexpressing Ptf1a and Rbpjl differentiated through-out the whole protocol. Staining was performed for Amyl (a) and Cpa1 (b) in red. Nuclei were stained in blue. Negative control (c) was performed with an irrelevant antibody. Scale bars: a–c, 10 µm. (TIF) [file pone.0054243.s003.tif]

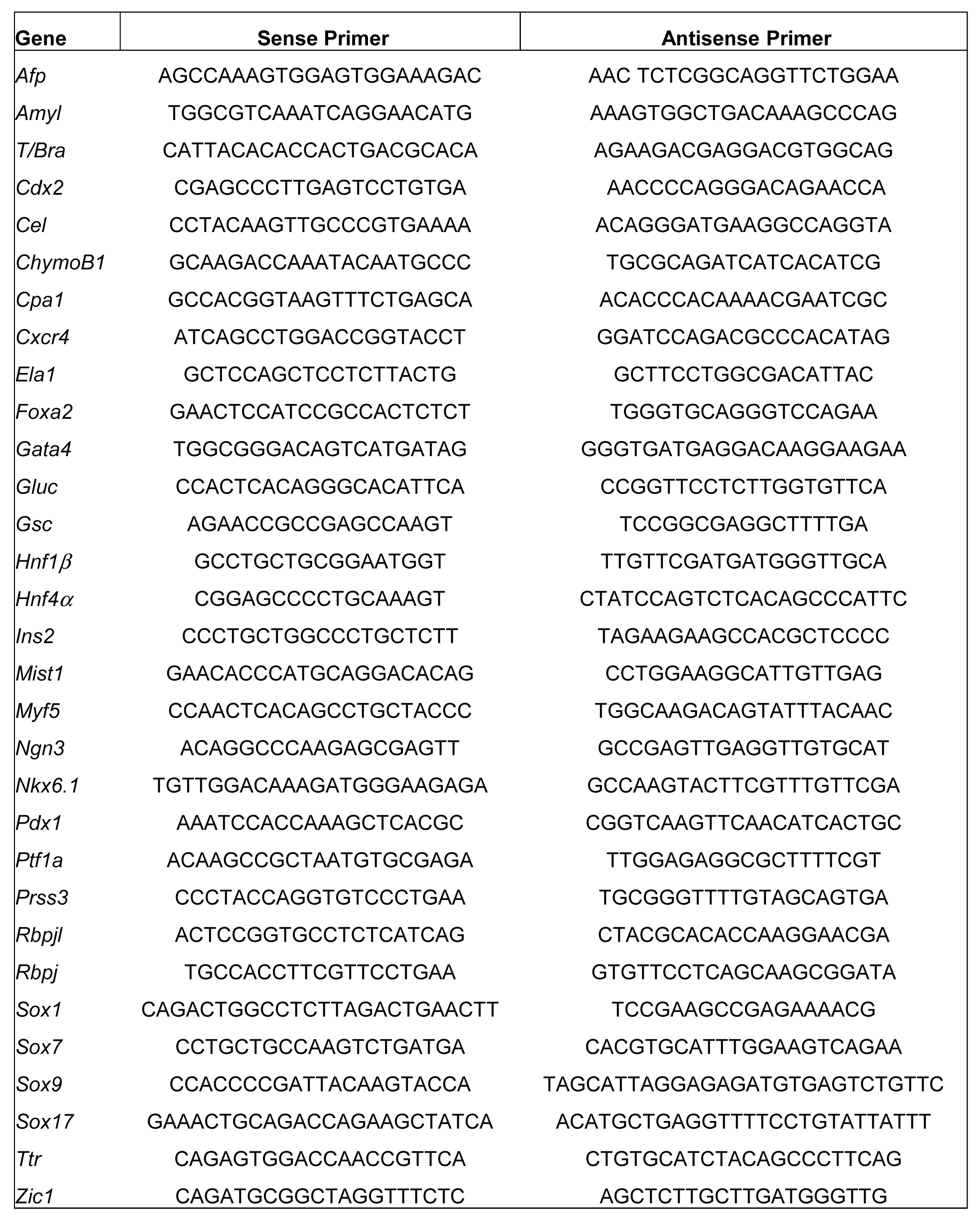

Supplement: Table S1 — List of primers used for qPCR. (TIF) [file pone.0054243.s004.tif]
